# Supplementary figures and images for: The impact of prediabetes on preclinical atherosclerosis in general apparently healthy population: A cross-sectional study
Source: PLoS One. 2024 Oct 29;19(10):e0309896. doi: 10.1371/journal.pone.0309896 (PMC11521245; doi:10.1371/journal.pone.0309896)

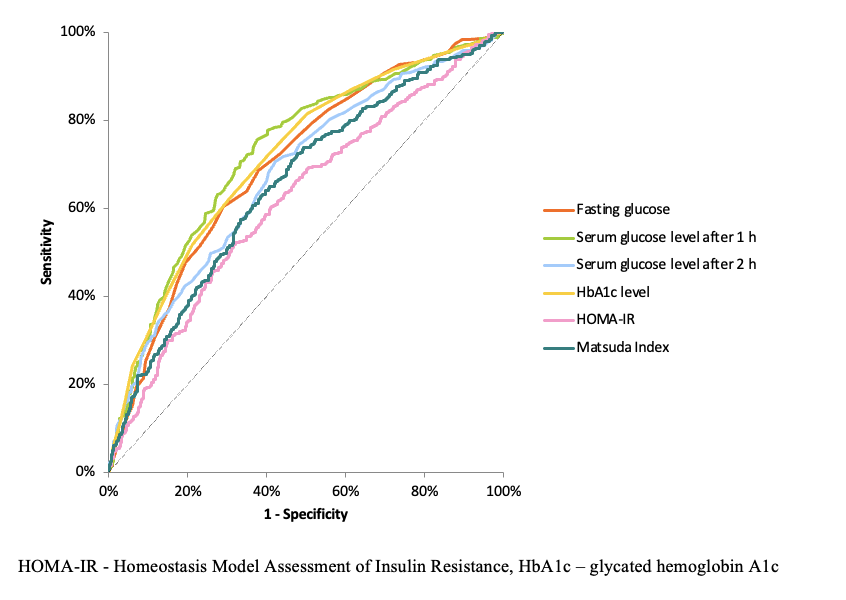

Supplement: S1 Fig — Dependent variable: the presence of any atherosclerotic plaques on ultrasound of the carotid arteries. (TIFF) [file pone.0309896.s002.tiff]

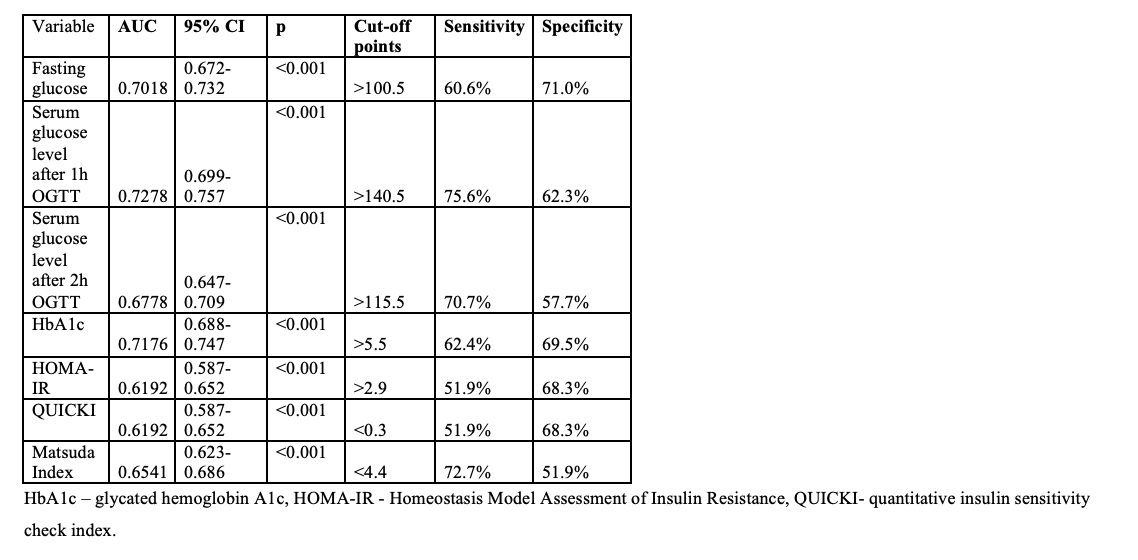

Supplement: S1 Table — (TIFF) [file pone.0309896.s003.tiff]

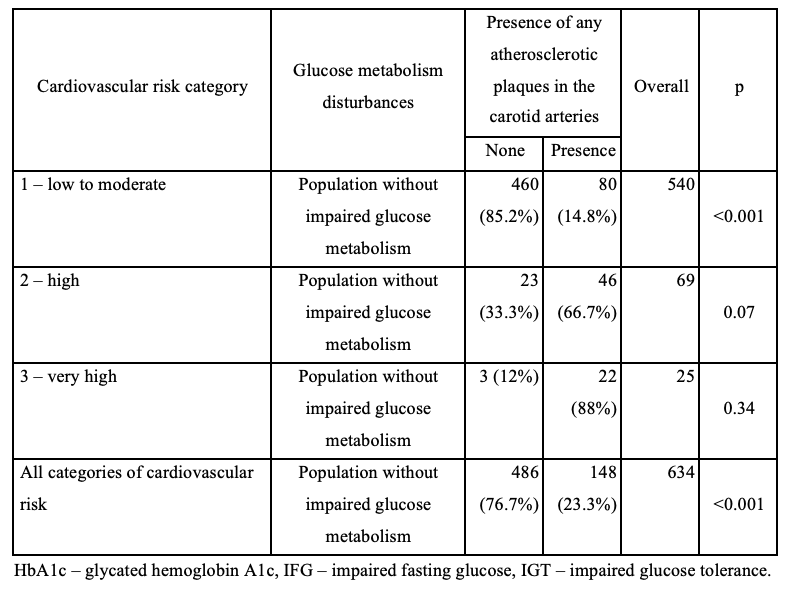

Supplement: S2 Table — P-values were derived from Chi-square tests. (TIFF) [file pone.0309896.s004.tiff]

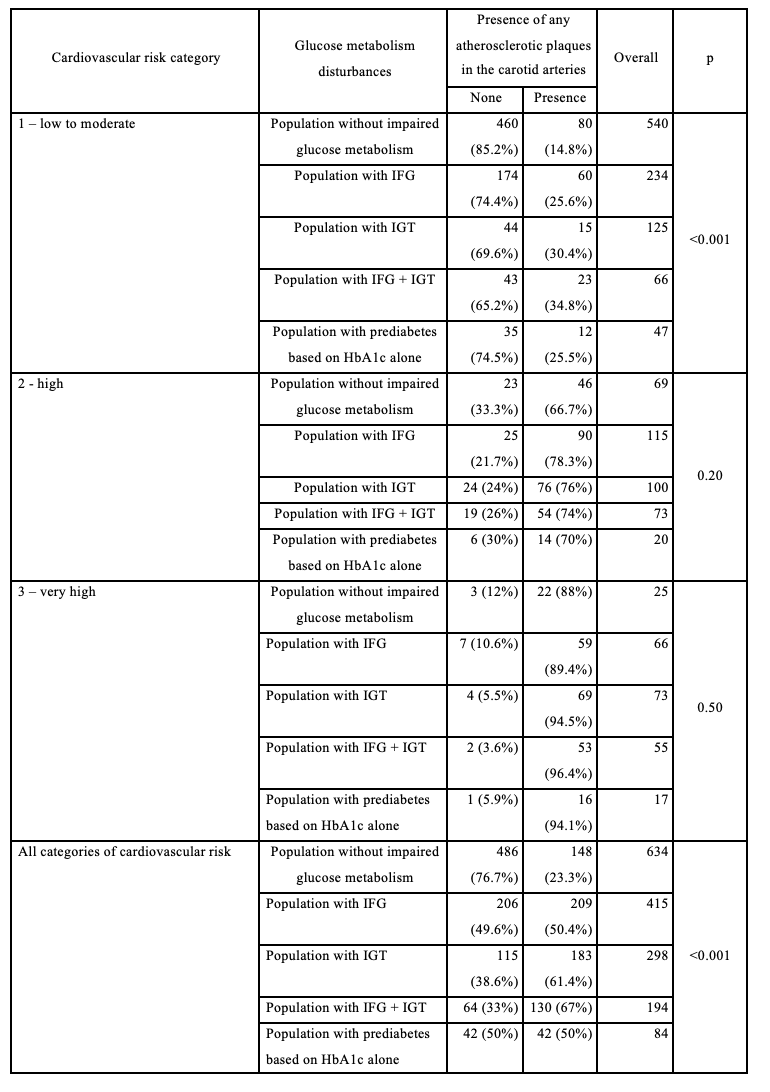

Supplement: S3 Table — P-values were derived from Chi-square tests. (TIFF) [file pone.0309896.s005.tiff]

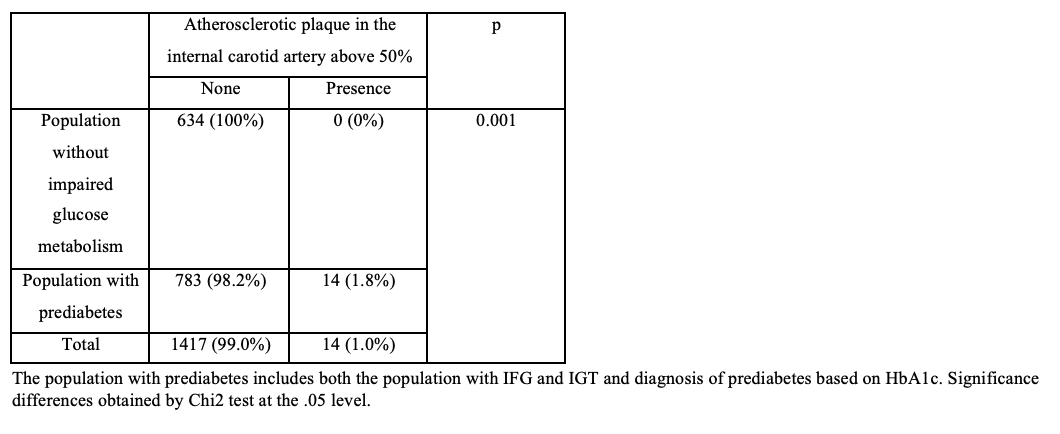

Supplement: S4 Table — (TIFF) [file pone.0309896.s006.tiff]

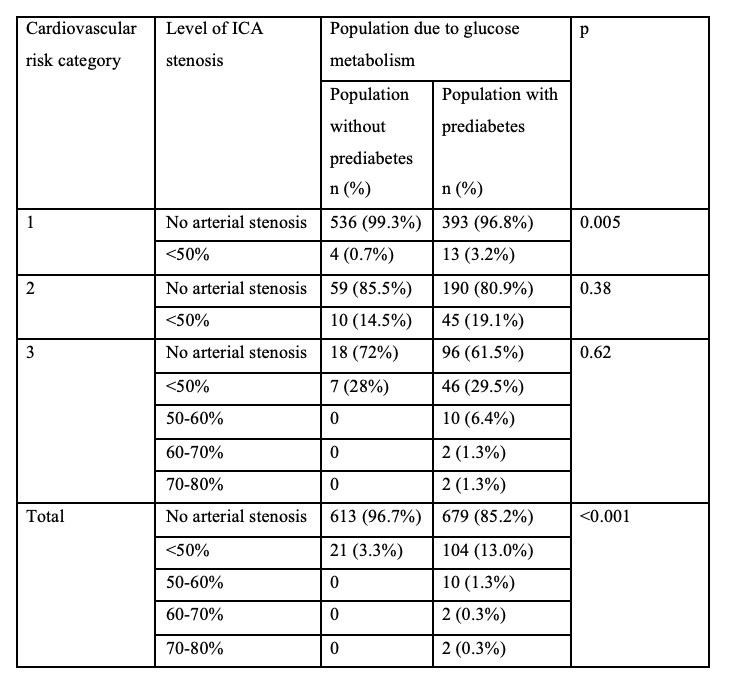

Supplement: S5 Table — P-values were derived from Chi-square tests. (TIFF) [file pone.0309896.s007.tiff]
