# Supplementary material for: The impact of prediabetes on preclinical atherosclerosis in general apparently healthy population: A cross-sectional study
Source: PLoS One. 2024 Oct 29;19(10):e0309896. doi: 10.1371/journal.pone.0309896 (PMC11521245; doi:10.1371/journal.pone.0309896)
Supplement: Attachment — Submitted filename: Response to Reviewers.docx [file pone.0309896.s008.docx]

17th February 2024

Professor Karol Kamiński

Department of Population Medicine and Lifestyle Diseases Prevention

Medical University of Białystok

Dear Editor

We would like to thank you for your helpful comments on the manuscript *The impact of prediabetes on preclinical atherosclerosis in general apparently healthy population.* We have edited the manuscript to address all the concerns. All the changes we have made are marked in red.

We hope that after corrections suggested by the Editorial Office, the manuscript is now suitable for publication in the PLOS ONE.

We are looking forward to hearing from you soon.

Yours sincerely

Karol Kamiński

On behalf of all authors.

**Comments from PLOS Editorial Office:**

**We apologize for the difficulties you were having with your submission. For your convenience, we have summarized the points previously raised by the academic editor below:**

**- Accept all changes and cancel tracking mode.**

The changes have been accepted. Tracking mode has been canceled.

**- Use a uniform font and font size throughout the manuscript including references, tables and figure legends.**

The font has been standardized to Times New Roman, font size 12.

**- Mention all tables (including the supplementary ones) in the main text and sort them according to their order of appearance in the text**

Tables and figures are mentioned in the main text of the manuscript.

**- Move the study limitations into the Discussion**

Study limitations have been moved to the last paragraph of the Discussion.

**- Put the Declarations part on a separate page and arrange it more clearly**

The declarations were placed on page 2 of the manuscript and prepared in accordance with the journal's recommendations.

**- Rearrange the figures and tables so that they are readable: In particular, the authors should put each item on a separate page and format it in a way so that it does not exceed this page.**

The figures were prepared in accordance with these rules. The tables have been reformatted according to this recommendation, but Tables 1 and 3 contains too much information to fit on one page.

**- Use no colours in the tables**

Well, it has been corrected.

**- Use "." as decimal separators in all tables**

Commas have been changed to dots.
