# Supplementary material for: The impact of prediabetes on preclinical atherosclerosis in general apparently healthy population: A cross-sectional study
Source: PLoS One. 2024 Oct 29;19(10):e0309896. doi: 10.1371/journal.pone.0309896 (PMC11521245; doi:10.1371/journal.pone.0309896)
Supplement: Attachment — Submitted filename: Response to Reviewers.docx [file pone.0309896.s009.docx]

June 18, 2024

Prof Karol Kamiński, MD PhD

Department of Population Medicine

and Lifestyle Disease Prevention

Medical University of Bialystok

e-mail: fizklin@wp.pl

Dear Editor

We would like to thank you for your helpful comments on the manuscript *The impact of prediabetes on preclinical atherosclerosis in general apparently healthy population: a cross-sectional study.* We have edited the manuscript to address all the concerns. All the changes we have made are marked in red.

We hope that after corrections suggested by the reviewer, the manuscript is now suitable for publication in the PLOS One Journal.

We are looking forward to hearing from you soon.

On behalf of all authors.

Yours sincerely

Karol Kamiński

**Additional Editor Comments:**
 **General:
- The authors should make sure that their manuscript follows the STROBE guidelines.**

Thank you for suggestion. We have corrected the manuscript accordingly and clearly stated this in the manuscript. We have created a revised Flowchart (Figure 1 Supplementary Material). We have described the population study method in detail. Moreover, our data has been recalculated and completed, due to numerous statistical comments.

**- Both the title and the abstract should mention the cross-sectional study design.**

I added in the title: *The impact of prediabetes on preclinical atherosclerosis in general apparently healthy population: a* ***cross-sectional study****.*

I added also in Abstract, Methods: *The cross-sectional analysis included 1431 participants of a population-based cohort study. Comprehensive biochemical assessments were performed.*

**- At some occasions, the results are presented in present tense. Instead, all results should be described in past tense.**

I have changed the tense as suggested.

**- The authors present a bunch of similar analyses on the same dataset, leading to issues of multiple testing. Preferably, only those results which are directly related to the main hypothesis should be shown, while several side analyses might just be left out. I added some suggestions below. Additionally, p-values and interpretations of statistical significance should be presented only for the most relevant associations.**

Thank you for your comment. We have made the corrections as suggested below.

**- In the spirit of Open and Reproducible Science, the analysis code should be made available in an online repository together with a data dictionary, and the respective URL should be mentioned in the Methods section.**

We did not used pre-programmed scripts. We have done ad hoc analysis in the SPSS.

**Abstract:
- The abstract should mention setting, locations, periods of recruitment, duration of follow-up, and age of the participants.**

The following sentence has been added:

*The analysis was a part of the Bialystok PLUS cohort study and represented a random sample of Bialystok residents aged 20-79 at the time of sampling (July 2017-January 2023).*

**- The sentence "In multivariate logistic regression, prediabetes significantly increased risk for preclinical atherosclerosis (p=0.015)." should be rephrased to "...was associated with significantly increased risk...", and OR with 95% CI should be shown instead of a p-value.**

Thank you, it has been corrected.

*In multivariate logistic regression, prediabetes was independently associated with significantly increased risk of preclinical atherosclerosis (OR= 1.56, 95% CI 1.09-2.24), along with CVR categories, pulse wave velocity and central blood pressure augmentation index.*

**- It seems not correct to state that the analysis included 1866 participants given that a large proportion of them were excluded due to technical reasons or prevalent DM, and it also does not fit to the statement ..."more than half of the study participants (n=797, 51.9%)"**

In accordance with the Editor's comments, we have included the correct flowchart of study population (Figure 1 Supplementary Materials) and changed the number given as size of the population to 1431.

*The cross-sectional analysis included 1431 participants of a population-based study (mean age 48.82).*

**Introduction:
- "...and 319 million adults (6.2%) with IFG" should be rephrased as "... to have IFG"
- When introduced, abbreviations should be used throughout the manuscript, which is e.g. not the case in the sentence "First, the presence of nondiabetic hyperglycemia signifies an increased risk of developing type 2 diabetes..."**

Both corrections have been applied.

**- The study hypothesis is not properly motivated. For example, the authors state that prediabetes is associated with a higher risk of cardiovascular disease. So would it not just be expected that it is also associated with a higher risk of preclinical atherosclerosis? Are there already any other studies on this topic?**

Indeed, the introduction was too brief. An additional paragraph has been added to further motivate the research hypothesis. It should be emphasized that there are quite strong controversies about the effect of prediabetes on atherosclerosis and hence cardiovascular risk. Currently European Society of Cardiology guidelines do not incorporate prediabetes in their cardiovascular risk calculators, mainly due to limited evidence from general population. Therefore the evidence our study provides contributes to this scientific discussion.

*A previously conducted study of patients with first-time acute coronary syndrome (ACS) who underwent urgent coronary angiography showed that coronary atherosclerosis is more advanced in patients with prediabetes than in patients without DM [38]. Furthermore, the association of the occurrence of coronary atherosclerosis was shown to be comparable between patients with prediabetes and patients with DM at the time of first ACS [38]. Another study using percutaneous coronary intervention showed that coronary lesions in prediabetic patients were associated with higher levels of lipid-rich atherosclerotic plaques [39]. Prediabetes is associated with inflammation and vasoconstriction, which promote atherosclerosis in the coronary arteries [40]. Notably, limited evidence comes from research on both, prediabetes and carotid atherosclerosis in general population.*

**Methods:
- In the sentence "We randomly sampled citizens in such numbers as to obtain a distribution of proportions in terms of age and gender similar to that of the city's population.", what is meant by "in such numbers"? Please add that this sentence only pertains to the age range mentioned before.**

The text has been transformed for better understanding.

*Our analysis was a part of the Bialystok PLUS cohort study and represented a random sample of Bialystok residents aged 20-79 at the time of sampling (July 2017-January 2023). Bialystok is a medium-sized city located in eastern Poland with a population of 293,400. The recruitment of participants for the population-based study uses a pseudonymized list of residents of Bialystok obtained from the Local Municipal Office. Annually, we randomly sampled citizens to obtain a distribution of proportions in terms of age and gender reflecting that of the city's population. A more detailed study design was described in previously published paper [11].*

**- What is meant by "research tests"?**

We meant all the tests performed in the course of the study, as they were performed solely for the purpose of the study. For greater clarity, we have changed the research test to “the clinical and biochemical measurements”.

*All the clinical and biochemical measurements were conducted by qualified medical personnel.*

**- In the Matsuda formula, a bracket seems to be missing. Can the abbreviations I0 and G0 also be used in the HOMA-IR and Matsuda formulas?**

Thank you for helpful suggestions. Corrections have been made in accordance with it.

*Quantitative insulin sensitivity check index (QUICKI) was defined as 1/[log(I_0_) + log(G_0_)], where I_0_ is the fasting insulin (μU/ml), and G_0_ is the fasting glucose (mmol/l) [16]. Homeostasis model assessment of insulin resistance (HOMA-IR) was calculated using the formula: I_0_  ×  G_0_ /22.5 [15]. When assessing insulin sensitivity, we used the Matsuda index calculated from the formula 10000/ √ (G_0_ mg/dl x I_0_ μU/ml) x (Mean OGTT glucose concentration mg/dl × mean OGTT insulin concentration mg/dl) [17].*

**- "... for checking variable normality." should be revised to "... for checking whether the variables followed a normal distribution."**

Done.

**- Supplementary Tables 1 and 2 seem superfluous, as these definitions can easily be mentioned in the main text in the methods section.**

The tables have been removed. A description has been added in *Methods, Assessment of Insulin sensitivity*.

**Results:
- Please add a descriptive table 1 to characterize the whole study population (excluding the participants excluded).**

A description of the whole study population has been added to Table 1, in the last column ‘Total’.

**- Supplementary Figure 2 seems superfluous, the more so as it is not mentioned in the main text.**

Indeed, according to this Supplementary Figure 2 has been deleted.

**- The current tables 1 and 3 are heavily overloaded and need to be condensed or split into two separate tables each (e.g. with and without prediabetes markers). The p-values should be removed, as they are sensitive to sample size and prone to multiple testing. Instead, I would suggest to show mean and 95% confidence intervals instead of median and IQR (see also the comments of Reviewer 1 concerning this).**

In accordance with this comment and the suggestions of Reviewer 1, all tables have been revised.

**- The sentence "We used the diagnostic criteria for DM and prediabetes..." seems out of place in the results section.**

It has been moved to *Methods* section.

**- The numbers in Suppl. Figure 1 do not add up to 1866. The authors should show a proper flowchart as requested in the STROBE guidelines (which would not include the numbers of participants with or without prediabetes).**

Figure 1 Supplementary Material has been modified.

**- Did the persons excluded due to technical reasons differ from the remaining population with respect to age, sex or other characteristics? If so, potential bias by exclusion of these participants needs to be discussed.**

| **Variable** | **Excluded population for technical reasons n=183**  **Mean, 95% CI** | **Study population**  **n=1431**  **Mean, 95% CI** | **Total**  **n=1866**  **Mean, 95% CI** |
| --- | --- | --- | --- |
| Age | 49.67 (47.49-51.85) | 46.82 (46.05-47.59) | 47.14 (46.41-47.87) |
| BMI | 27.29 (26.24-28.33) | 26.30 (26.06-26.54) | 26.39 (26.15-26.62) |
| WHR | 0.85 (0.84-0.87) | 0.86 (0.85-0.86) | 0.86 (0.85-0.86) |
| SBP | 118.08 (115.10-121.06) | 122.18 (121.31-123.04) | 121.85 (121.02-122.68) |
| DBP | 79.56 (77.73-81.39) | 80.48 (79.96-80.99) | 80.40 (79.91-80.90) |
| Total cholesterol | 196.56 (188.78-204.33) | 193.63 (191.59-195.67) | 193.86 (191.89-195.84) |
| LDL-C | 124.30 (117.27-131.32) | 123.86 (122.00-125.72) | 123.89 (122.09-125.69) |
| Fasting glucose | 97.38 (95.55-99.21) | 97.87 (97.38-98.36) | 97.83 (97.35-98.30) |
| HbA1c | 5.42 (5.35-5.49) | 5.36 (5.34-5.38) | 5.36 (5.34-5.38) |
| Sex, %male | 29.0% (22.9%-35.9%) | 44.9% (42.4%-47.5%) | 43.1% (40.7%-45.6%) |
| The presence of atherosclerotic plaque | 43.0% (34.7%-51.6%) | 40.7% (38.2%-43.2%) | 40.9% (38.4%-43.3%) |
| Cholesterol-lowering treatment | 75.6% (65.8%-83.3%) | 78.7% (75.4%-81.7%) | 78.3% (75.2%-81.2%) |

In the table we have included a comparison between those excluded for technical reasons (n=183) and the analysed population (n=1431). The values of the parameters we were able to compare are not considerably different from each other. We believe that, despite the statistical difference, the compared populations do not differ clinically.

**- "The population of attendees without dysglycemia consisted of 634 patients (41.2%). Importantly, the population with prediabetes accounted for more than half of the study participants (n=797, 51.85%)." Why do these two numbers not add up to 100%? As a minor point, percentages should presented with the same number of digits throughout the whole manuscript (main text and tables).**

Percentages have been rounded to 1 decimal places in the texts. Previously, the percentages were counted to the whole group (including those who were excluded). For a better understanding of the analysis, changes have been made.

*In further analysis, we considered 1431 participants. The population of attendees without dysglycemia consisted of 634 patients (44.3%). The population with prediabetes accounted for more than half of the study participants (n=797, 55.7%).*

**- "Importantly, the population without glucose metabolism disturbances": Leave out "Importantly" (there should be no interpretation in the results section)**

Thank you.

**- What is meant by "a statistically relevant difference"?**

We have changed to “*statistically significant difference”.*

**- All supplementary tables should be named by the order of their appearance in the main text.**

Done.
**- The abbreviation USG should be explained in the main text and each figure / table where it appears.**

Done.
**- The legends of Figure 1 and Supplementary Table 5 should not be selective in mentioning the AUC values of the predictors, i.e. show all or none of them.**

OK, the AUC values have been removed from the legends.

**- "most statistically significant indicators" is wrong wording. A result can either be statistically significant or not.**

Indeed. This has been corrected.

*We observed that all examined parameters proved to be statistically significant indicators of the presence of atherosclerotic plaques in carotid ultrasound, also upon application of Bonferroni correction. However, the highest AUC values were obtained for serum glucose concentration after 1h and HbA1c (AUC=0.73; 95% CI 0.70-0.76 for glucose level after 1 h and AUC=0.72; 95% CI 0.69-0.75 for HbA1c).*

**- Why does Supplementary Table 5 not include glucose level after 1 h, and why is the AUC of HbA1c different in the table to the AUC mentioned in the main text?**

Many thanks for your thorough review of the manuscript. Table 5 Supplementary Materials has been corrected. The values entered in the Manuscript were correct. This was a mistake in transferring data from a statistical programme to a word document.

**- Table 2 is difficult to read and might rather be replaced by e.g. stacked barplots.**

Table 2 has been transformed into stacked barplots.

**- It is confusing that some tables are arranged with CVR as columns and glucose metabolism and rows and vice versa. The appearance of these tables should be unified.**

We wanted to emphasise what exactly is being compared in the tables by using this approach. These are additional analyses and have therefore been placed in Supplementary Materials.

**- Supplementary Tables 4 and 12: The p** values should be removed.**

Done.

**- The logistic regression analyses are confusing and contradictory in their results, which is particularly irritating given that there seem to be quite strong associations between prediabetes and preclinical atherosclerosis in bivariate analyses. The results from Supplementary Table 8 seem to be based on the most reasonable approach (while the other regression analyses seem superfluous), but they indicate a negative association between the two factors. Can the authors please comment on this issue?**

To sum up, bivariate analyses suggest that there are positive associations between prediabetes and preclinical atherosclerosis. In the multivariate analyses, we used two separate approaches. In the first multivariate model, we included individual classical CVR factors (age, sex, SBP, total cholesterol, LDL-C, cigarette smoking, BMI) as co-variates. In this model, we did not show a statistically significant association between prediabetes and the occurrence of atherosclerotic plaques, which may be due to strong association of prediabetes with other risk factors (age, blood pressure, BMI) and the fact that they may have a strong association with preclinical plaques. In the second approach, we used a cardiovascular risk scale considering the cardiovascular risk factors in a synthetic way proposed by the European Society of Cardiology. In this model, we present an independent positive relationship (Table 10, Supplementary Materials) between prediabetes and the presence of preclinical carotid artery lesions (95% CI 1.091-2.232, OR 1.561), which emphasizes potential value of including prediabetes in cardiovascular risk assessment. Model 2 informs us that the prediabetes in addition to CVR categories is associated with a higher risk of preclinical atherosclerosis, which may be relevant for physicians in clinical practice.

**Discussion:

- The authors applied a large number of tests, but did not correct for multiple testing. This should be clearly stated in the discussion together with the consequence that their results should be seen as hypothesis-generating and need confirmation from other studies.**

Thank you for this comment. In our case, we tested the hypothesis whether prediabetes increases the risk of preclinical atherosclerosis. Additional analyses were hypothesis generating and assessed the mechanism of this association. A Bonferroni correction was applied to the ROC analysis as suggested.

- "This study confirms a very high prevalence of prediabetes in a general population." The statements in this paragraph and also in the conclusion are too general and need to be more specific to the underlying population. Further, the authors might discuss how generalizable their results are with respect to other populations in Europe and across the world.

**Thank you very much for this comment. The prevalence of impaired glucose metabolism was the topic of our previous paper, which is cited in this article as *31. Zieleniewska NA, Szum-Jakubowska A, Chlabicz M, Jamiołkowski J, Kowalska I, Kamiński KA. The prevalence of diabetes and prediabetes: a population-based study. Pol Arch Intern Med. 2023 Jan 5:16407. doi: 10.20452/pamw.16407. Epub ahead of print. PMID: 36602858.* In this paper we focus on the link of prediabetes and preclinical atherosclerosis. I have removed this conclusion on the basis that it may indeed be confusing.**

**Reviewer #1: This manucript examines the impact of prediabetes on preclinical atherosclerosis in general apparently healthy population in Poland. The paper is well written and organised.**

Please find my comments below:

**- Statistical analysis section: It is stated that continuous variables are described using medians (IQR). However, in Table 1, it seems that some variables (i.e. Age) are described using mean +/- sd. This should be clarified in the statistical analysis section as well as the results for this.**

Statistical analysis section was described again, considering the changes made to the manuscript.

*Descriptive statistics for continuous variables were presented as means and 95% Confidence Intervals for means and for categorical variables as proportions and 95% Confidence Intervals for proportions. The sample size is large enough to assume that the variables followed a normal distribution according to Central Limit Theorem.*

**- Statistical analysis section: Shapiro-Wilks test is sensitive to large sample sizes and the null hypothesis can be rejected even with small deviations from normality. Kolmogorov-Smirnov is a better option when sample size is >50. However, here the sample size is quite large so the central limit theorem holds. The authors could have proceeded with using only parametric tests.**

As you mentioned, the sample size is large enough to assume that sample means are approximately normal distributed according to Central Limit Theorem. Therefore, we used parametric tests in statistical analysis. Modifications have been made to all tables and the description of results.

**- Fig.2: SPSS 27 does not produce forest plots. The authors should state clearly the software used for the data analysis and visualisation.**

Forest plots were prepared in Excel based on coefficients calculated in SPSS 27. We added information about that in the Manuscript.

*The figures have been made in Excel and R.*

**- Any p-values=0.000 should be changed to <0.001.**

Indeed, this has been changed.

**Reviewer #2: The manuscript entitled "The impact of prediabetes on preclinical atherosclerosis in an apparently healthy general population" addresses a highly interesting and significant clinical issue. Early identification of individuals with dysglycemia is essential for the effective implementation of cardiovascular and metabolic prevention measures. Considering the authors' understanding of the research problem, the reviewer requests a discussion on the practical application of the obtained results - whether this data is sufficient for updating/altering recommendations, particularly for General Practitioners, and what those changes should involve?**

Our findings may have implications for the management of patients with preclinical conditions such as prediabetes and asymptomatic carotid atherosclerosis. Our study suggests that expansion of outpatient care in higher-risk patients who are not included in the risk calculators proposed in the recommendations. SCORE-2 does not include prediabetes and thus may underestimate the risk of preclinical atherosclerosis. Our results support this conclusion, as prediabetes significantly contributes to the risk of atherosclerosis when analyzed with CVR classes and provides additional pivotal information. Once prediabetes, especially IGT, is diagnosed, the GP should suggest lifestyle changes and increased physical activity and intensify follow-up visits to observe the development of preclinical atherosclerosis.

**Reviewer #3: The paper PONE-D-23-42492R1 » The impact of prediabetes on preclinical atherosclerosis in general apparently healthy population« describes the relation of prediabetes (diagnosed by fasting glucose levels or by oral glucose tolerance test) and the presence of carotid artery plaques in a random sample of 1866 subjects from the Bialystok PLUS study. Prediabetes significantly increased risk for preclinical carotid atherosclerosis according to a multivariate logistic regression model.
I have the following comments:

Materials and methods, Assessment of insulin sensitivity. Please, list the criteria for diagnosing prediabetes.**

Thank you for the suggestion. We have included the criteria used to diagnose prediabetes in Methods, Assessment of insulin sensitivity as suggested. Table 2, Supplementary Materials have been deleted as it contained the same information.

*We used the diagnostic criteria for DM and prediabetes according to ESC and the European Association for the Study of Diabetes (EASD) recommendations [1]. Diabetes was not diagnosed based on fasting glucose due to availability of a single measurement. Prediabetes was diagnosed in participants who had no previous diabetes: 1) IFG if fasting glycaemia was between 100-125 mg/dl (5.6-6.9 mmol/l) and after 2h was less than 140 mg/dl (7.8 mmol/l); 2) IGT if at 2h glycaemia was ≥140 mg/dl (7.8 mmol/l) and < 200 mg/dl (11.1 mmol/l); 3) based on HbA1c when it ranged from 5.7 - 6.4 % (42-47 mmol/mol). Those who had fasting glucose in a single measurement above 125 mg/dl and after 2h below 200 mg/dl were categorised as IGTs. Newly diagnosed DM was diagnosed based on OGTT ≥ 200 mg/dl and an HbA1c ≥ 6.5%.*

**Typographical corrections:

Abstract, line 9: Atherosclerotic plaques in the carotid arteries were …

Fig. 1. The x-axis should be labeled »1-specificity«.**

Thank you, both mistakes have been corrected.
