# Supplementary material for: The impact of prediabetes on preclinical atherosclerosis in general apparently healthy population: A cross-sectional study
Source: PLoS One. 2024 Oct 29;19(10):e0309896. doi: 10.1371/journal.pone.0309896 (PMC11521245; doi:10.1371/journal.pone.0309896)
Supplement: Attachment — Submitted filename: Response to Reviewers.docx [file pone.0309896.s010.docx]

July 16th, 2024

Prof Karol Kamiński, MD PhD

Department of Population Medicine

and Lifestyle Disease Prevention

Medical University of Bialystok

e-mail: fizklin@wp.pl

Dear Editor

We would like to thank you for your comments on the manuscript *The impact of prediabetes on preclinical atherosclerosis in general apparently healthy population: a cross-sectional study.* We have edited the manuscript to address all the concerns. All the changes we have made are marked in red.

We hope that after corrections suggested by the reviewer, the manuscript is now suitable for publication in the PLOS One Journal.

We are looking forward to hearing from you soon.

On behalf of all authors.

Yours sincerely

Karol Kamiński

**Journal Requirements:

Please review your reference list to ensure that it is complete and correct. If you have cited papers that have been retracted, please include the rationale for doing so in the manuscript text, or remove these references and replace them with relevant current references. Any changes to the reference list should be mentioned in the rebuttal letter that accompanies your revised manuscript. If you need to cite a retracted article, indicate the article’s retracted status in the References list and also include a citation and full reference for the retraction notice.**

The list has been checked. All references are correct and have not been changed or retracted.

**Additional Editor Comments:

The authors have improved their manuscript considerably. Only a few points remain to be addressed:

General:
- There are still some results described in present tense, e.g. those under "Atherosclerotic plaque presence in particular cardiovascular risk categories". The whole manuscript should be checked and revised again regarding this issue.**

Indeed, the tense used has been corrected.

**- Throughout the whole manuscript (including tables and figures), only black font should be used.**

Done.

**- Please re-check and confirm that all numbers mentioned in the main text fit to the numbers presented in the tables and figures.**

Done. **Abstract:
- Please add "years" after 20-79 and 48.82 (also in the main text), and mention that Bialystok is a city from Poland.**

Done.

**- Please omit "the" in "proved to be the statistically significant indicators".**
Thank you, corrected.

**Results:
- Figure 1 should be moved from the main document to the Supplement, and vice versa for Supplementary Figure 1 (flowchart).**

Done.

**- Suggest to show vertical bars in Figure 2 and to remove the yellow bars (sorry, my previous comment hinting to a "stacked barplot" was somewhat misleading).**

Done.

**- Suggest to move the "Total column" in Table 1 to the very left.**

Done.

**- "Additionally, 166 patients were excluded from further analysis for technical reasons...". This number cannot be found in the flowchart. Please explain.**

Thank you, it was a typing mistake. Everything now corresponds with Flowchart.

The study enrolled 1866 participants. According to STROBE guidelines, the flowchart represents the study population (Figure 1 Supplementary Materials). Prevalent diabetes (n=252, 13.5%) was diagnosed on the basis of medical history (n=140) and glucose levels after 2h OGGT (n=112). All prevalent diabetic patients were excluded from further analysis. Additionally, 183 patients were excluded from further analysis for technical reasons – inability to draw blood, perform an OGTT or US of the carotid arteries [31]. After applying these exclusion criteria, the data of 1431 participants remained for analysis.

**- Suggest to revise "In further analysis we considered 1431 participants." by e.g. "After applying these exclusion criteria, the data of 1431 participants remained for analysis."**

Ok.

**- It makes no sense to present the Supplementary Tables 5 and 6 as they are likely to be prone to collinearity issues as mentioned in the text. Instead, the choice (and exclusion) of the predictors in Supplementary Table 7 should be properly motivated and described in detail, possibly accompanied by a stepwise variable selection process. The predictor prediabetes should be shown at the top of this table. Why were sex and age not included here?**

That's fine, in that case we deleted Table 5 and Table 6 Supplementary Materials. Gender and age were not included because they are already included in the cardiovascular risk categories. We used SCORE calculators to calculate CVR categories, which take gender and age into account. We decided to exclude these variables to avoid potential multicollinearity problem in regression model. We have changed the description in the main text.

*We also performed multivariate logistic regression model where the dependent variable was the presence of any atherosclerotic plaques (Table 3). We built a model in which we included CVR categories (which already include classic cardiovascular risk factors, including gender and age), glucose metabolism and vascular stiffness parameters. Figure 2 Supplementary Materials is a graphical summary of the fully adjusted logistic regression model. Prediabetes was associated with significantly increased risk of preclinical atherosclerosis (OR= 1.56, 95% CI 1.09-2.24; p=0.014), along with CVR categories, pulse wave velocity and central blood pressure augmentation index.*

**- Finally, it would seem more appropriate to move Supplementary Table 7 to the main results and to remove Figure 3 instead.**

Done.

**Reviewer #1: The authors have replied to my comments adequately. I only have one minor comment. Please indicate the dependent variable in the captions of Tables 5 and 6.**

Thank you. According to the editor's comments, these tables have been deleted from the supplement. We added caption in Table 3 of the main text considering the dependent variable.

**Reviewer #3: The manuscript is now substantially improved. The reviewers' comments have been adequately addressed.**

Thank you.
