# Supplementary material for: The impact of prediabetes on preclinical atherosclerosis in general apparently healthy population: A cross-sectional study
Source: PLoS One. 2024 Oct 29;19(10):e0309896. doi: 10.1371/journal.pone.0309896 (PMC11521245; doi:10.1371/journal.pone.0309896)
Supplement: Attachment — Submitted filename: Response to Reviewers.docx [file pone.0309896.s011.docx]

July 25, 2024

Prof Karol Kamiński, MD PhD

Department of Population Medicine

and Lifestyle Disease Prevention

Medical University of Bialystok

e-mail: fizklin@wp.pl

Dear Editor

We would like to thank you for your comments on the manuscript *The impact of prediabetes on preclinical atherosclerosis in general apparently healthy population: a cross-sectional study.* We have edited the manuscript to address all the concerns. All the changes we have made are marked in red.

We hope that after corrections suggested by the reviewer, the manuscript is now suitable for publication in the PLOS One Journal.

We are looking forward to hearing from you soon.

On behalf of all authors.

Yours sincerely

Karol Kamiński

**Additional Editor Comments:**

**The manuscript is now almost ready for journal acceptance. Please accept all changes made so far so that only changes required for this (hopefully last) revision are clearly visible. The following points remain to be addressed:
- Unit "years" is still lacking behind "20-79" in the Abstract, in the Results and in Figure 1.**

Done. **- Any results which are mentioned in the Abstract should also be mentioned in the main text (e.g. mean age).**

Done.

**- The interpretation that prediabetes was independently associated with preclinical atherosclerosis is very important for the main message of this paper and should therefore also be mentioned in the results section and explained how this conclusion was arrived at (i.e. that this association remained significant after adjustment for confounders). I would also suggest to move prediabetes above CV risk in table 3 to make this result more visible. It might also be worth to mention in the text how the OR of preclinical atherosclerosis by prediabetes would look like without adjustment for the confounders mentioned in table 3.**

**Prediabetes was moved at the top of Table 3. We also added in the Results section:**

Based on these results, we conclude that prediabetes was independently associated with preclinical atherosclerosis. This association remained significant after adjustment for confounders and the final findings were shown in Table 3.

**- It would be helpful to mention all the numbers from the flowchart also in the main text to explain to the Reader how the number of 183 patients who were excluded from further analysis for technical reasons was achieved.**

We have changed to “Additionally, 183 patients were excluded from further analysis for technical reasons – inability to perform an OGTT (n=132) or US of the carotid arteries (n=4) or lack of the HbA1c measurement (n=7) or lack of CVR assessment (n=40)”.

**- Figure 1: "n=7 subjects were exclused" should read "excluded", and "USG" be replaced by "US".**

Done.

**- Please mention which version of R was used.**

Done.

**- In all tables including the supplement, "C.I." should be replaced by "CI" for reasons of consistency.**

Done.

**- In table 3, the p-values should be mentioned to the right of the 95% CIs.**

Done.

**Further, as the supplementary figures and tables will probably not be quality-checked after acceptance, it is important that they fulfil the necessary standards right now. Please see specific comments below:
- Please add "Supplementary" before each figure and table.**

Done.

**- The supplementary tables should be ordered in the way they are mentioned in the text (i.e. supplementary table 1 should be placed between supplementary tables 4 and 5).**

Changed.

**- Asteriks should be removed from the p-values, all p-values >0.01 should be presented with 2 decimals.**

Done **- Each table should start on a new page.**

Done

**- In supplementary table 2, the sentence "* there is a statistically significant difference after Bonferroni correction." should be replaced by "P-values were calculated using Bonferroni correction for the number of variables investigated (n=7)".**

Done.

**- In supplementary tables 3 and 5, the sentence "Significance differences obtained by Chi2 test at the .05 level." should be replaced by "P-values were derived from Chi-square tests." and put into the figure legend (not in the footnote).**

Done.

**- Which test was applied in supplementary table 4?**

Chi2 test was applied. I added in the caption of Supplementary table 4.

**- Combined columns such as in supplementary tables 3 (All participants with prediabetes) and 4 (Total) should be avoided.**

Done.

**- Supplementary table 5 still contains coloured font. "p* Differences between population with or without prediabetes in a particular CVR category." should be removed.**

Done.

**- Supplementary figure 2: This Figure might be removed altogether. If the authors decide to keep it, the following changes should be considered: The title "Model: Full" is not informative. "Logistic regression models" in the legend is confusing, as the ORs seem to have been derived from only one model. The values on the x-axis should be presented in horizontal direction. It should be mentioned that the values on the x-axis follow a logarithmic scale. Suggest to remove the OR of serum insulin level from this plot.**

Thank you. We decided to remove this figure.
